# Supplementary material for: Prevalence of undernutrition and associated factors among street adolescents in adama town, oromia regional state, Ethiopia, 2023: A cross sectional study
Source: PLoS One. 2024 Jan 2;19(1):e0296500. doi: 10.1371/journal.pone.0296500 (PMC10760772; doi:10.1371/journal.pone.0296500)
Supplement: Attachment — Submitted filename: point by point response for PLOS ONE.docx [file pone.0296500.s001.docx]

Title: Prevalence of undernutrition and associated factors among street adolescents in Adama town, Oromia regional state, Ethiopia, 2023: A Cross sectional study, PONE-D-23-26853

From: Authors

### To: PLOS ONE

Version: 1

Point -by-point response by authors

| Sections | Independent Review Report |  | Response to comments by Authors |
| --- | --- | --- | --- |
|  | Reviewer #1 |  |  |
| General comments on the manuscript | Thank you for your important research project Nonetheless, good to respond adequately and clearly to the following inquiries: |  | Dear respected reviewer, we thank you for your genuine comments. The comments are helpful to increase the quality our study. |
| introduction | a) What is the research gap you wanted to bridge in your study as it is universally understood that most street adolescents in different towns of the country who are disadvantaged both economically and socially are likely to be significantly undermentioned.? b) What makes your study different from similar studies conducted previously at different towns including Jimma town at Oromia region in Ethiopia? |  | a)-Thank you for your comments, as far as we know there is no study done on adolescent undernutrition in Adama; In Ethiopia, government offices and different civil organizations are working in collaboration in different cities to overcome all the problems that all street adolescents face. To assist street children and reduce the number of children living on the street, the Social Service Society Children's Village (SOS) has also started a project in the three major cities Ethiopia; namely, Addis Abeba, Adama, and DireDawa. Our intension is therefore, to highlight and show the outcomes of the effort made by those organization in Adama town and to point out additional remedial actions that must be carried out by the responsible bodies based on the current findings.  b) Obtaining recent evidences for possible comparison of the outcomes in different part of the country can bring a significant impact for action as well. |
| Methods  -Sample size calculation  -Sampling technique  - data collection | c) In Adama town as you reported there were over 5000 street adolescents and of them, your sample size 358 street children were identified using convenience sampling method . Do you think that they are adequate enough to represent the source population?  d) In your community based cross sectional study how do you explain the validity and generalizability of your findings obtained from convenience sampling method?.  - g) As you know the street adolescents are very unstable population those move from place to place very frequently, how did you limit their very mobility during your data collection period ?  h) As the main aim of a public health important research is for action or interventions, how do you explain the feasibilities of your recommendations related to street children in today’s Ethiopia? |  | C)-Thank you so much respected reviewers, our sample size is relatively good in comparison with the previous study conducted in other part of the country and it was calculated based on the prevalence obtained before. Even if the Bureau of Women's, Children's, and Youth’s Affairs in Oromia collaborated with UNICEF tries to count the number of street children in Adama town, the real figure in each kebele is not clearly determined due to their living slandered (street adolescents are very unstable population those move from place to place very frequently) which makes difficult to apply probability sampling techniques and possible allocation in each kebele for representation.  -d) generalizability issues as we mentioned above is therefore, highlighted as limitation  .  g)-right, to avoid such misshaping we have used indelible ink/dye which was applied on the forefingers of the study participants as semi-permanent/cannot be erased in short period of time.  h)- as we have mentioned above, there is an organization working on it in collaboration with the government. Therefore, showing the current situation of this population with evidence as scholars may help them for possible remedial action early in time. |
| Ethics | e) As over 75 % of your study participants were minors, with no guardians or parents in your study setting, how did you solve the challenges of obtaining written consents as well as securing assents to the acceptable level of ethical considerations? |  | e)- Informed written consent to participate was obtained from all participants and parents of minor participants. For those street adolescents without parents, we received written informed consent from them after the Administration's Labor and Social Affairs Office approached us to provide assent for that adolescent's participation in research which was also approved by the institutional review board (IRB) of Jimma University Institute of Health for ethical approval. |
| Result | f) In Ethiopian context where by in many instances the living cost is sky rocketing , did you expect most street children to have a good nutritional status; to get daily meals without forcibly skipping ; to have a clean water to drink; to maintain a good personal hygiene ,etc while you are selecting your independent variables ? |  | -right, almost for all, the current situation of the country is challenging and the fore mentioned study participant were the most vulnerable population in all aspects. however, if the government works aggressively with other stakeholders in this regard, we can minimize and even eliminate the devastating living situation of such special underprivileged populations in the country; and expected to have good nutritional status in this population as well. |
| recommendation | h) As the main aim of a public health important research is for action or interventions, how do you explain the feasibilities of your recommendations related to street children in today’s Ethiopia? |  | h)- as we have mentioned above, there is an organization working on it in collaboration with the government. Therefore, showing the current situation of this population with evidence as scholars may help them for possible remedial action early in time. |
|  | Reviewer #2: |  |  |
| General Comments | Dear author/s, thank you so much for your works manuscript entitled “Prevalence of undernutrition and associated factors among street adolescents in adama town, oromia regional state, Ethiopia, 2023: A Cross sectional study’. I have some comments and suggestions; |  | Thank you! Respected reviewers for your genuine comments. |
| Abstract | It is not recommended not use abbreviations in abstract. Please, write key words based on Mesh. |  | -Thank you! Respected reviewers for your genuine comments, now it has been corrected. |
| introduction | Rewrite the paragraph 2 of introduction with its citations.. |  | -okay, thank you! Corrected! See the revised manuscript. |
| Sample size calculation | Please, make clear your sample size calculation and write the single population proportion formula. Why you do not calculate sample size for associated factors? |  | Thank you so much respected reviewers, and we use this single population proportion formula; this is because it yields relatively larger sample sizes; We have calculated the sample size by using double proportion formula and unfortunately, all yields smaller than the one calculated by using single population proportion formula.  . |
| Sampling procedure and sampling technique | Sampling procedure and sampling technique is not clear. By what method you choose kebeles? Why you choose convenience sampling technique? Is the calculated sample size proportionally allocated to each selected kebeles? Did you get list of street adolescents? 5000 adolescents? |  | - Thank you so much respected reviewers, Even if the Bureau of Women's, Children's, and Youth’s Affairs in Oromia collaborated with UNICEF tries to count the number of street children in Adama town, the real figure in each kebele is not clearly determined due to their living slandered (street adolescents are very unstable population those move from place to place very frequently) which makes difficult to apply probability sampling techniques and possible allocation in each kebele for representation; and that is why we have used convenience to select both kebeles and study participants. Based on the information obtained from the Bureau of Women's, Children's, and Youth’s Affairs, concentrated street adolescents were observed in the fore mentioned kebele there. |
| data collection | Under data collection please collect this sentences “BMI was calculated by weight in kg divided by height squared in cm.” |  | Thanks! It is type error….we use meter squared |
| data processing and analysis | Did you use different version of SPSS? In abstract it says SPSS V25 and in data processing and analysis is SPSS V26. |  | Thank you once again! It is also type error ;we have used SPSS version 25. |
| Results | How did you 100% response rate? Please, rewrite result part (remove ambiguous word and redundancy of words). |  | -Thank you, the nature of our sampling method enables it to be 100% in terms of response rate; the other issue of result writing is also corrected! see the revised manuscript. |
|  | Reviewer #3 |  |  |
| General comment | This paper addresses a long existing issue of undernutrition in street adolescents. The use of primary data collection methods is notable. The conclusions are consistent with the results but needs more evidence to justify some recommendations made by the authors. The paper needs to be proofread and edited. Despite the authors’ attempt to address the important issue of undernutrition, there are concerns from introduction to the conclusion section in this study. |  | Noted, thank you so much for your valuable comments! |
| introduction | The introduction should be condensed to a maximum of 2 pages. For instance, paragraphs 2 and 3 have almost the same idea in different paragraphs. Similarly, paragraphs 1 and 4 are discussing the same idea in different paragraphs. So, use connectors and condense them into the same paragraph. Here are also some grammatical problems to be corrected. Unnecessary upper case, but necessary under case. Correct them.  In the last paragraph of the introduction, the author tried to state the knowledge gap as there has not been enough research, particularly in Adama Town. Do you think it is feasible to conduct research in each town in the country? A number of studies have been conducted on street children, specifically on nutrition. Make your study knowledge gap stronger by reviewing the gap in other ways. |  | -The introduction part of the manuscript is now condensed based on your recommendation. See the revised manuscript.  -The grammar issue is also evaluated and addressed by fluent English language experts.  -As far as we know there is no study done on adolescent undernutrition in Adama; most of the studies conducted before in other part of the country was also focused on children not on adolescents; In Ethiopia, government offices and different civil organizations are working in collaboration in different cities to overcome all the problems that all street adolescents face. To assist street children and reduce the number of children living on the street, the Social Service Society Children's Village (SOS) has also started a project in the three major cities Ethiopia; namely, Addis Abeba, Adama, and DireDawa. Our intension is therefore, to highlight and show the outcomes of the effort made by those organization in Adama town and to point out additional remedial actions that must be carried out by the responsible bodies based on the current findings.  b) Obtaining recent evidences for possible comparison of the outcomes in different part of the country can bring a significant impact for action as well. |
| Methods: Sample size and Sampling procedures | It is known that Adama City has a sub-city. But you didn’t mention that in your sampling procedure. Clarify the reason. How did you decide on five kebele? Clarify the procedure. Please describe the procedure using a flow chart. You used the convenience sampling technique without explaining the reason. Please explain that. There are streets off and streets on children. You included both. Don't you think that they have different interesting outcomes? How did you manage this? |  | -- Thank you so much respected reviewers, Even if the Bureau of Women's, Children's, and Youth’s Affairs in Oromia collaborated with UNICEF tries to count the number of street children in Adama town, the real figure in each kebele is not clearly determined due to their living slandered (street adolescents are very unstable population those move from place to place very frequently) which makes difficult to apply probability sampling techniques and possible allocation in each kebele for representation; and that is why we have used convenience to select both kebeles and study participants. Based on the information obtained from the Bureau of Women's, Children's, and Youth’s Affairs, concentrated street adolescents were observed in the fore mentioned five kebeles there. |
| operational definition | Your outcome variable operational definition is not clear. You said it was either stunting or thinness. Contrary to this, your dependent variable is stated as the prevalence of undernutrition (stunting and thinness), which needs more clarity. |  | Thank you so much, it is to mean that, stunting or thinness or both together in one corresponding study participant |
| result | The overall prevalence of undernutrition was 169 (47.2%). Include the confidence interval. Even though it is clear how the author measured stunting and wasting, but it is not clear how you measured The overall prevalence of undernutrition Operationalize it objectively. There is a repetition of the results in the conclusion section of the study. Try to summarize your findings in a different manner than repeating the result. About figure 1 and 2 The author presented stunting and thinness using a bar graph, which accounted for 122 (34.1%) and 73 (20.4%), respectively, and 26 (7.3%) of both stunting and thinness. A Venn diagram could be useful in presenting this. |  | -Thank you once again, we have modified based on the recommendation. See the revised manuscript highlighted with track change.  -The graph presentation style is also corrected based on your comments. |
